# Effects of different selenium sources and levels on antioxidant status in broiler breeders

**DOI:** 10.5713/ajas.18.0226

**Published:** 2018-05-31

**Authors:** K. X. Li, J. S. Wang, D. Yuan, R. X. Zhao, Y. X. Wang, X. A. Zhan

**Affiliations:** 1 Key Laboratory of Animal Nutrition and Feed in East China, Ministry of Agriculture and Key Laboratory of Animal Feed and Nutrition of Zhejiang Province, Feed Science Institute, College of Animal Science, Zhejiang University, Hangzhou 310058, China; 2 College of Animal Science and Technology, Zhejiang A and F University, Linan 311300, China

**Keywords:** Sodium Selenite, Selenium-enriched Yeast, Selenomethionine, Broiler Breeders, Antioxidant Status

## Abstract

**Objective:**

This study was conducted with the objectives to examine the impacts of inorganic selenium (Se) and different types and levels of organic selenium on the serum and tissues Se status and antioxidant capacity in broiler breeders.

**Methods:**

Five hundred and forty 48-wk-old Lingnan Yellow broiler breeders were randomly assigned to 6 dietary treatments, provided same basal diet (0.04 mg/kg of Se) with 0.15 mg/kg, or 0.30 mg/kg of Se from sodium selenite (SS) or from selenium-enriched yeast (SY) or from selenomethionine (SM). The broiler breeders were slaughtered after an 8-wk experiment.

**Results:**

The results showed that SM was better than SY and SS, 0.30 mg/kg level was better than 0.15 mg/kg level in Se deposition (p<0.05) in serum, liver, kidney, pancreas and muscle; in antioxidant status, organic selenium had better effects than SS in broiler breeders (p<0.05), but SM and SY had a similar result, and 0.15 mg/kg level was better than 0.30 mg/kg (p<0.05).

**Conclusion:**

The results demonstrated the evident advantage of supplementation of broiler breeders with 0.15 mg/kg SM, which improved tissue Se concentrations and antioxidant status, and can be considered as the best selenium source.

## INTRODUCTION

Selenium (Se) has been recognized as an essential trace element involved in resistance to oxidative stress [1]. Rotruck reported that Se was a necessary ingredient in glutathione peroxidase (GPx), which was identified as a very potent antioxidant protecting the body from damage due to oxidation by free radicals [2]. As one of the main components, Se mainly achieved antioxidant physiological function through GPx and other selenoproteins. Supplementation with Se is a common practice in broiler production. In general, the sources of Se added into poultry diet can be categorized into two forms: organic Se and inorganic Se. Dietary Se supplementation of chickens improved feed conversion and weight gain compared to control group without Se supplementation [3]. In addition, several studies have pointed out that organic forms of Se have excellent bioavailability and are appropriate for nutritional supplementation [4–6]. Besides, organic forms of Se are less toxic than inorganic forms, such as sodium selenite (SS) [7,8]. It has been found that dietary Se increased the Se concentrations in the liver, kidney and muscle of broilers, while Se yeast (SY) produced higher concentrations than SS [9,10]. Dalia et al [11] also reported that broiler chickens receiving bacterial organic Se had higher tissue Se deposition and better antioxidant status [11]. What’s more, Se accumulation and GPx activity of broiler breeders were higher in the group fed 0.30 mg/kg SY compared to 0.30 mg/kg of SS [12].

However, the chemical forms of Se in SY are very complex. At present, we have very limited knowledge of the data related to the content and composition of selenium yeast. SY contains inorganic forms of Se such as selenium acid, selenium and organic forms of Se such as selenomethionine (SM), selenocystine, selenohomocysteine, methylselenol, and trimethylselenonium. Importantly, SM has the highest biological potency in the body. Moreover, DL-Se-Met was approved as a feed additive for all animal species on August 4th, 2014 by the European commission. And on October 19th, 2015, L- Se-Met could be produced and used in China. So far, no studies have reported the difference between SY and SM in broiler breeders. Therefore, this study was conducted with the objectives to examine the effects of different selenium sources and levels on selenium concentrations and antioxidant capacity in broiler breeders.

## MATERIALS AND METHODS

This project was approved and conducted under the supervision of the Zhejiang University Animal Care and Use Committee, which have adopted Animal Care and Use Guidelines governing all animal use in experimental procedures. All efforts were made to minimize suffering.

### Birds, diets, and study design

Five hundred and forty 48-wk-old Lingnan Yellow broiler breeders were randomly divided into 6 dietary treatments, each of which was replicated 6 times with 15 birds per replicate. After a four-week period feeding basal diet to deplete body reserves of Se, the broiler breeders were fed the same basal diet (0.04 mg/kg of Se) supplemented with 0.15 mg/kg of Se, 0.30 mg/kg of Se from SS (Control group) (Sigma-Aldrich Chemical Co., St. Louis, MO, USA) or from selenium-enriched yeast (Sel-Plex, Alltech Inc., Nicholasville, KY, USA) or from SM (Sigma-Aldrich Chemical Co., USA) for another 8 wk. The basal diet was formulated to meet the nutrient requirements of broiler breeders according to NRC (National Research Council) [13] except for Se (Table 1). The birds were housed in laying battery cages, 2 hens per cage in the same house and provided with clean water and fed ad-libitum during the experimental period.

After the last week of the experiment, 4 hens were randomly selected per replicate from each treatment and starved for 12 h before slaughter, and blood samples were taken from the main wing vein. A 10.0-mL sample was extracted per bird and let coagulate at room temperature for 1 h. The serum was separated by centrifugation at 4°C, 1,000 g for 20 min and transferred into 1.0-mL microcentrifuge tubes. After blood collection, the birds were killed and dissected. The fresh right pectoralis major muscle per bird was collected for meat analyses. Tissues of liver, kidney, pancreas, and breast muscle were dissected carefully, blotted free of blood and placed in liquid nitrogen. All were stored at −80°C for further analysis.

### Selenium analyses

The Se assay of the serum and tissue samples was performed by hydride generation atomic fluorescence spectrometry [14]. The 0.2 mL of serum and 0.2 g tissue samples were digested in a digestion tube using microwave digestion system with 1.0 mL H_2_O_2_. Deionized water was added to the remaining digests to produce a 10.0 mL solution after 7 minutes. Then 2.0 mL filtered digests, 1 mL of 50% hydrochloric acid, 1.0 mL mixture of 5% sulfuric acid and 5% ascorbic acid were added in 10.0 mL test tube, after 15 min of reaction, then kept the 10.0 mL system by adding deionized water. The Se concentration was measured immediately using the dual-channel atomic fluorescence spectrometer [15,16].

### Biochemical assays

Tissue samples were thawed at 4°C for 30 min. Then the samples were immediately homogenized in an Ultra-Turrax (T8, IKA-Labortechnik, Staufen, Germany) for 10 s at 8,000 rpm with 9 volumes of homogenization buffer (0.86% sodium chloride). This process was conducted on ice. The homogenate was centrifuged at 4,000 rpm for 10 min at 4°C and the supernatant was collected for analysis. The GPx, total superoxide dismutase (T-SOD), catalase (CAT), total antioxidation capability (T-AOC), malondialdehyde (MDA) and protein concentration were measured in serum, liver, kidney, pancreas, and muscle tissues. Antioxidant enzymes analysis was performed using kits (Nanjing Jiancheng Bioengineering Company, Jiangsu, China) according to the manufacturer’ instructions [17,18].

### Statistical analysis

All data are presented as mean±standard deviation and assessed by SPSS 19.0 software. Compare the main effect of selenium source and level by general linear model. Least significant difference method was used to compare the significant difference data. Values of p<0.05 were considered significance level.

## RESULTS

In this study, organic Se supplemented groups significantly increased the Se concentrations in serum, liver, pancreas and muscle in the broiler breeders (p<0.05) compared with the SS. In the kidney, SM supplemented group had greater Se concentration than others, but the SS and SY groups showed no significant difference (p>0.05). Besides, SM supplemented group had significantly increased Se concentrations in serum and kidney, but did not differ with liver, pancreas and muscle (p>0.05). Compared with the 0.15 mg/kg Se level, the 0.30 mg/kg Se level had the higher Se concentration. Samples of serum and liver had an interaction between Se sources and levels (p<0.05) (Table 2).

Se sources and levels had no significant effect on GPx activity in serum and in the studied organs (p>0.05). The 0.15 mg/kg group had the higher GPx activity than the 0.30 mg/kg group (p<0.05), but were close between the two treatments in liver, kidney and muscle (p>0.05). Moreover, 0.15 mg/kg Se supplementation level tended to increase the GPx activity of liver in broiler breeders. There was also an interactive effect on GPx activity in the kidney between the Se sources and levels (p<0.05) (Table 3).

Total antioxidation capability activity in liver and kidney rose due to the supplementation of SM (p<0.05) (Table 4). There was no significant difference between SS and SY (p> 0.05). In addition, the Se sources showed no significant difference in T-AOC activity in serum, pancreas and muscle (p> 0.05). T-AOC activity in serum, liver and muscle was significantly higher than 0.30 mg/kg after dietary 0.15 mg/kg Se supplementation (p<0.05). But the Se levels had no significant effect for T-AOC activity in kidney and pancreas (p>0.05). In the pancreas and muscle, the Se sources and levels had an interaction effect on T-AOC activity (p<0.05).

Table 5 shows that SM significantly improved T-SOD activity in liver (p<0.05) and had no significant correlation with SS (p>0.05). Besides, there was no significant difference of T-SOD activity among the Se sources in serum, kidney, pancreas and muscle (p>0.05). The 0.15 mg/kg selenium supplementation group had a higher T-SOD activity than 0.30 mg/kg in the kidney (p<0.05). But in serum, liver, pancreas and muscle, the Se supplementation levels showed no significant correlation on T-SOD activity (p>0.05).

As is shown in Table 6, SM and SY significantly improved CAT activity in liver (p<0.05) compared with SS. There was no significant difference in CAT activity in liver between SM and SY (p>0.05). The SM supplementation group had a higher CAT activity than SS (p<0.05). Besides, the Se sources did not significantly alter CAT activity in serum, pancreas and muscle (p>0.05). Furthermore, the Se levels did not produce differences in CAT activity in either serum or in studied organs (p>0.05). In liver, the Se sources and levels had interaction effect on CAT activity (p<0.05).

Compared with SS and SY, SM significantly decreased MDA concentration in kidney (p<0.05) (Table 7). And MDA concentration of SY and SM was lower than SS in muscle after dietary organic Se (p<0.05). However, the Se sources had no significant effect on MDA concentration in serum, liver and pancreas (p>0.05). Besides, 0.15 mg/kg significantly reduced MDA concentration in serum and tissues compared with 0.30 mg/kg except in liver (p<0.05) (Table 7).

## DISCUSSION

It is known that organic Se has a higher deposition efficiency than inorganic Se [19–22]. In the present study, compared with SS, there was a huge increase of Se concentrations in serum and in the studied organs caused by SM and SY supplementation, which was in good agreement with findings of Pan et al [23] and Leeson et al [24]. There were two possible reasons: organic Se has different metabolic pathways compared to inorganic Se; Se containing proteins have a better ability to be concentrated in some tissues [25]. In addition, compared with SY, SM can significantly improve the Se concentrations in serum and kidney. This was probably because SM induced a higher Se concentration than SY. Another result in this study showed that 0.30 mg/kg Se supplementation significantly improved the concentrations of Se in serum and in the studied organs compared to 0.15 mg/kg.

Among all varieties of selenoproteins, GPx was first discovered member of the best known ones, which play a critical role in antioxidative defense in poultry [26,27]. It is widely distributed in the body to protect the structure and function of cell membranes by catalyzing toxic peroxides into non-toxic hydroxyl compounds and promoting the decomposition of H_2_O_2_. Because the activity center of GPx is selenocysteine, it is generally believed that GPx activity can reflect the selenium levels in the body. Petrovic et al [28] reported that dietary Se supplementation could significantly increase GPx activity of serum, liver, kidney and duodenal mucosa of laying broiler breeders. Placha et al [29] recently found that broiler diets containing Se can improve GPx activity of serum and liver. Many studies have shown that a certain level of selenium in the body was necessary for GPx activity. And in our study, SM and SY could significantly improve GPx activity, but there was no significant difference between SM and SY.

The T-AOC is a comprehensive index for measuring the antioxidant system function in the body. A low T-AOC activity indicates a state of oxidative stress or oxidative damage in the body. SOD and CAT are the first oxidative barrier against free radicals in the body and are of vital importance for removal of free radicals and maintaining cell energy metabolism. SOD can repair and restore damaged cells, through catalyzing the disproportionation reaction of the superoxide anion (O^2−^) (2O^2−^+2H^+^→H_2_O_2_+O_2_) and block cell damage caused by reactive oxygen species (ROS). CAT has a similar biological function with GPx, catalyzing hydrogen peroxide to water and oxygen. However, a molecule of CAT can catalyze millions of molecules of hydrogen peroxide per second which is different from other enzymes. In this study, we found that SM can significantly improve the activity of T-AOC in breeders’ liver and kidney compared with SS and SY. Previous studies [30] also reported that, dietary organic selenium resulted in a higher activity of T-AOC in the broiler pectoral muscle than inorganic selenium. Moreover, SM dietary supplementation can significantly improve the activity of T-SOD, CAT in liver and CAT in kidney. This is supported by the finding of Ahmad et al [30] who found dietary supplementation of organic selenium can significantly improve the activity of T-SOD and CAT in broiler pectoral muscle compared to inorganic selenium. Pan et al [31] reported that supplemented Se for laying hens can significantly improve the SOD activity in plasma.

Lipid peroxidation is the most important oxidative stress caused by oxygen free radicals. MDA, an important indicator of lipid peroxidation, is one of the final products of cell polyunsaturated fatty acid peroxidation [32,33]. It can show the degree of radical attack, lipid peroxidation level and the ROS metabolic status. In this experiment, SM significantly decreased MDA concentration in kidney and muscle compared with SS and SY, which were similar with Devore et al [34]. The same results were obtained by Ahmad et al [30], who reported that organic selenium supplementation diet had less MDA concentration than inorganic selenium. It might be SM has a higher bioavailability than SS and SY, thus raising the antioxidants levels and decreasing the production of lipid peroxidation products.

## CONCLUSION

To summarize, our study showed that SM was better than SY and SS, 0.30 mg/kg level was better than 0.15 mg/kg level in selenium deposition; in antioxidant status, organic selenium had a better effect than inorganic selenium in broiler breeders, but SM and SY had a similar result, and 0.15 mg/kg level was better than 0.30 mg/kg. It was shown that the optimum selenium source was 0.15 mg Se/kg SM.

## Figures and Tables

**Table 1:** Composition and nutrient levels of broiler breeder basal diets (g/kg, unless otherwise stated)

Items	
Ingredients	
Maize	646.0
Soybean meal, 43% CP	250.0
CaHPO_4_	18.0
Limestone	70.0
Salt	3.0
DL-methionine	3.0
Premix1)	10.0
Nutrient levels	
ME2) (MJ/kg)	11.2
Crude protein	161.1
Calcium	30.2
Total phosphorus	6.5
Lysine	8.2
Methionine	5.5
Met+Cys	8.1

CP, crude protein; ME, metabolizable energy.

1) Supplied the following per kilogram of diet: vitamin A, 10,800 IU; vitamin D_3_, 2,160 IU; vitamin E, 27 IU; vitamin B_12_, 0.01 mg; folic acid, 1.08 mg; biotin, 0.18 mg; menadione, 1.4 mg; thiamin,1.8 mg; riboflavin, 8 mg; pyridoxine, 4.1 mg; niacin, 32 mg; calcium pantothenate,11 mg; iron, 72 mg; copper, 7 mg; zinc, 72 mg; manganese, 90 mg; iodine, 0.9 mg.

2) ME was calculated from data provided by Feed Database in China.

**Table 2:** Effects of different sources and levels of selenium on selenium retention of broiler breeders

Item	Se amount (mg/kg)	Serum (mg/L)	Liver (mg/kg)	Kidney (mg/kg)	Pancreas (mg/kg)	Muscle (mg/kg)
SS	0.15	0.619±0.032^e^	0.420±0.018^c^	0.399±0.060^c^	0.191±0.021^d^	0.104±0.012^c^
	0.30	0.740±0.074^c^	0.481±0.031^b^	0.446±0.049^bc^	0.224±0.026^c^	0.125±0.009^b^
SY	0.15	0.681±0.037^d^	0.494±0.031^b^	0.426±0.053^c^	0.223±0.022^c^	0.119±0.008^bc^
	0.30	0.859±0.045^b^	0.608±0.026^a^	0.489±0.046^b^	0.261±0.015^ab^	0.164±0.027^a^
SM	0.15	0.703±0.017^cd^	0.510±0.056^b^	0.451±0.046^bc^	0.238±0.023^bc^	0.125±0.014^b^
	0.30	0.953±0.056^a^	0.643±0.049^a^	0.586±0.078^a^	0.268±0.047^a^	0.155±0.017^a^
SS		0.679±0.084^c^	0.451±0.040^b^	0.443±0.058^b^	0.208±0.028^b^	0.114±0.015^b^
SY		0.770±0.100^b^	0.551±0.065^a^	0.458±0.058^b^	0.242±0.027^a^	0.141±0.030^a^
SM		0.828±0.135^a^	0.576±0.085^a^	0.519±0.093^a^	0.253±0.039^a^	0.140±0.022^a^
	0.15	0.668±0.462^b^	0.475±0.054^b^	0.425±0.055^b^	0.217±0.029^b^	0.116±0.014^b^
	0.30	0.850±0.105^a^	0.577±0.079^a^	0.507±0.083^a^	0.251±0.036^a^	0.148±0.025^a^
p value	Se source	0.000	0.000	0.000	0.000	0.000
	Se amount	0.000	0.000	0.000	0.000	0.000
	Se source×amount	0.002	0.029	0.076	0.898	0.116

a–d Means (n = 12) within a column lacking a common superscript differ (p<0.05).

**Table 3:** Effects of different sources and levels of selenium on glutathione peroxidase activity in serum and tissues of broiler breeders

Item	Se amount (mg/kg)	Serum (U/mL)	Liver (U/mg prot)	Kidney (U/mg prot)	Pancreas (U/mg prot)	Muscle (U/mg prot)
SS	0.15	1,465±235^a^	24.79±3.23^a^	16.67±1.21^ab^	18.36±3.58^a^	2.46±0.62^a^
	0.30	1,064±170^b^	22.63±3.13^ab^	14.74±2.18^b^	18.20±3.23^bc^	1.16±0.63^b^
SY	0.15	1,383±97^a^	22.89±1.98^ab^	16.46±1.64^ab^	20.37±3.56^ab^	1.83±0.52^ab^
	0.30	1,179±118^b^	21.98±3.49^ab^	16.96±2.11^ab^	19.51±2.81^d^	1.77±0.67^ab^
SM	0.15	1,512±196^a^	21.24±1.97^b^	15.35±1.27^b^	22.17±5.30^d^	1.54±0.46^b^
	0.30	1,136±232^b^	22.66±2.73^ab^	17.95±2.81^a^	15.85±3.52^cd^	1.73±1.36^ab^
SS		1,265±286	23.71±3.23	15.71±1.96	18.28±3.23	1.81±0.90
SY		1,281±149	22.44±2.75	16.71±1.82	19.94±3.09	1.80±0.57
SM		1,324±284	21.95±2.39	16.65±2.49	19.01±5.41	1.64±0.97
	0.15	1,453±185^a^	22.97±2.75	16.16±1.43	20.30±4.28	1.94±0.64
	0.30	1,126±178^b^	22.42±2.96	16.55±2.63	17.85±3.38	1.55±0.93
p value	Se source	0.640	0.560	0.382	0.301	0.831
	Se amount	0.000	0.059	0.552	0.562	0.136
	Se source×amount	0.264	0.106	0.028	0.301	0.055

a–d Means (n = 12) within a column lacking a common superscript differ (p<0.05).

**Table 4:** Effects of different sources and levels of selenium on total antioxidation capability in serum and tissues of broiler breeders

Item	Se amount (mg/kg)	Serum (U/mL)	Liver (U/mg prot)	Kidney (U/mg prot)	Pancreas (U/mg prot)	Muscle (U/mg prot)
SS	0.15	7.90±2.00^a^	1.09±0.14^ab^	8.24±0.52^bc^	0.52±0.10^b^	0.54±0.08^a^
	0.30	4.86±0.99^c^	0.96±0.12^bc^	7.37±0.70^c^	0.89±0.33^a^	0.37±0.02^b^
SY	0.15	7.89±1.82^a^	1.13±0.12^a^	8.09±0.75^bc^	0.76±0.11^ab^	0.55±0.08^a^
	0.30	6.24±1.56^bc^	0.90±0.06^c^	8.48±0.61^ab^	0.92±0.34^a^	0.37±0.05^b^
SM	0.15	6.97±1.26^ab^	1.20±0.08^a^	9.04±1.25^ab^	0.88±0.37^a^	0.44±0.05^b^
	0.30	6.73±1.67^ab^	1.12±0.15^a^	9.39±1.34^a^	0.65±0.12^ab^	0.39±0.07^b^
SS		6.38±2.19	1.03±0.14^b^	7.81±0.74^b^	0.71±0.30	0.46±0.11
SY		7.06±1.85	1.02±0.15^b^	8.28±0.38^b^	0.84±0.26	0.46±0.11
SM		6.85±1.43	1.16±0.12^a^	9.21±1.25^a^	0.76±0.29	0.41±0.06
	0.15	7.59±1.71^a^	1.14±0.12^a^	8.46±0.94	0.72±0.26	0.51±0.09^a^
	0.30	5.94±1.59^b^	0.99±0.14^b^	8.41±1.23	0.82±0.29	0.38±0.05^b^
p value	Se source	0.466	0.008	0.003	0.455	0.166
	Se amount	0.001	0.001	0.887	0.246	0.000
	Se source×amount	0.054	0.332	0.180	0.026	0.022

a–c Means (n = 12) within a column lacking a common superscript differ (p<0.05).

**Table 5:** Effects of different sources and levels of selenium on total superoxide dismutase activity in serum and tissues of broiler breeders

Item	Se amount (mg/kg)	Serum (U/mL)	Liver (U/mg prot)	Kidney (U/mg prot)	Pancreas (U/mg prot)	Muscle (U/mg prot)
SS	0.15	156.8±8.0	192.2±20.0abc	110.8±10.8^a^	138.5±24.6	40.39±4.8
	0.30	156.8±7.8	189.7±21.4^bc^	105.7±14.8^ab^	154.0±37.9	38.85±4.1
SY	0.15	154.1±3.6	183.2±11.2^bc^	111.6±9.8^a^	170.0±21.8	38.11±4.0
	0.30	156.6±3.8	173.0±13.1^c^	97.7±11.8^b^	166.8±43.5	38.31±3.8
SM	0.15	160.3±8.8	210.5±14.4^a^	112.9±8.4^a^	173.7±85.0	41.57±3.8
	0.30	160.4±5.2	195.8±13.8^ab^	110.1±5.0^a^	131.9±15.6	41.56±5.1
SS		156.8±7.6	190.9±19.8^ab^	108.3±12.6	146.2±31.5	39.62±4.3
SY		155.3±3.8	178.1±12.8^b^	104.7±12.7	168.4±32.9	38.21±3.7
SM		160.3±7.0	203.2±15.5^a^	111.5±6.7	152.8±62.2	41.57±4.3
	0.15	157.1±7.3	195.3±18.8	111.8±9.1^a^	160.7±52.0	40.02±4.2
	0.30	157.9±5.9	186.2±18.4	104.5±11.9^b^	150.9±35.7	39.57±4.3
p value	Se source	0.096	0.003	0.292	0.465	0.173
	Se amount	0.658	0.099	0.047	0.511	0.756
	Se source×amount	0.830	0.644	0.403	0.289	0.862

a–c Means (n = 12) within a column lacking a common superscript differ (p<0.05).

**Table 6:** Effects of different sources and levels of selenium on catalase activity in serum and tissues of broiler breeders

Item	Se amount (mg/kg)	Serum (U/mL)	Liver (U/mg prot)	Kidney (U/mg prot)	Pancreas (U/mg prot)	Muscle (U/mg prot)
SS	0.15	1.98±0.21	31.74±7.50^b^	29.25±3.77^ab^	28.30±2.70	28.35±1.20
	0.30	1.85±0.49	28.80±4.38^b^	27.82±3.77^c^	31.80±7.40	29.00±1.40
SY	0.15	2.04±0.30	37.38±13.08^ab^	28.60±2.47^ab^	32.40±7.70	31.30±5.20
	0.30	1.94±0.55	49.98±10.02^a^	30.03±1.17^ab^	25.10±5.30	30.20±3.75
SM	0.15	2.12±0.37	51.72±26.28^a^	32.24±4.94^ab^	29.00±4.79	28.60±2.50
	0.30	2.03±0.72	31.98±9.84^b^	33.41±6.89^a^	30.30±6.70	31.25±7.35
SS		1.91±0.36	30.24±6.06^b^	28.47±3.64^b^	30.10±5.60	28.65±1.30
SY		1.99±0.43	43.68±12.90^a^	29.38±1.95^ab^	28.70±7.40	30.75±4.35
SM		2.07±0.55	41.82±21.54^a^	32.76±5.72^a^	29.70±5.60	29.95±5.40
	0.15	2.04±0.29	40.26±18.60	30.03±3.90	29.90±5.50	29.45±3.45
	0.30	1.94±0.57	36.90±12.48	30.42±4.94	29.00±6.80	30.15±4.65
p value	Se source	0.633	0.049	0.043	0.855	0.486
	Se amount	0.447	0.468	0.782	0.665	0.616
	Se source×amount	0.991	0.026	0.664	0.084	0.559

a–c Means (n = 12) within a column lacking a common superscript differ (p<0.05).

**Table 7:** Effects of different sources and levels of selenium on malondialdehyde concentration in serum and tissues of broiler breeders

Item	Se amount (mg/kg)	Serum (nmol/mL)	Liver (nmol/mg prot)	Kidney (nmol/mg prot)	Pancreas (nmol/mg prot)	Muscle (nmol/mg prot)
SS	0.15	3.41±0.28^c^	0.86±0.53^ab^	0.64±0.11^b^	1.77±0.72	3.00±0.71^bc^
	0.30	4.76±0.61^ab^	0.92±0.34^ab^	0.78±0.11^a^	1.99±1.09	3.98±0.79^a^
SY	0.15	4.19±0.52^bc^	1.18±0.27^a^	0.63±0.09^b^	1.57±1.01	2.36±0.46^cd^
	0.30	5.22±1.23^a^	0.66±0.29^b^	0.80±0.16^a^	1.82±0.80	3.36±0.72^ab^
SM	0.15	3.80±0.98^c^	1.00±0.31^ab^	0.54±0.06^b^	1.01±0.56	2.20±0.46^d^
	0.30	5.27±0.87^a^	1.18±0.35^a^	0.64±0.08^b^	2.40±0.96	3.03±0.61^bc^
SS		4.08±0.83	0.89±0.43	0.71±0.12^a^	1.88±0.89	3.49±0.88^a^
SY		4.71±1.05	0.92±0.38	0.71±0.12^a^	1.69±0.88	2.87±0.78^b^
SM		4.54±1.17	1.09±0.33	0.59±0.08^b^	1.71±1.04	2.61±0.68^b^
	0.15	3.80±0.71^b^	1.01±0.39	0.60±0.09^b^	1.45±0.81^b^	2.52±0.63^b^
	0.30	5.08±0.93^a^	0.92±0.38	0.74±0.13^a^	2.07±0.93^a^	3.46±0.78^a^
p value	Se source	0.092	0.343	0.009	0.844	0.006
	Se amount	0.000	0.436	0.000	0.042	0.000
	Se source×amount	0.727	0.052	0.707	0.195	0.944

a–d Means (n = 12) within a column lacking a common superscript differ (p<0.05).
